# Outcomes of the conservative management of the patients with endometrial intraepithelial neoplasia/endometrial cancer: Wait or treat!

**DOI:** 10.3906/sag-2012-207

**Published:** 2021-08-30

**Authors:** Esra İŞÇİ BOSTANCI, Yasin DURMUŞ, A.Sinem DURU ÇÖTELİ, Fulya KAYIKÇIOĞLU, Nurettin BORAN

**Affiliations:** 1 Department of Gynecologic Oncology, Faculty of Medicine, Gazi University, Ankara Turkey; 2 Department of Gynecologic Oncology, Mersin City Training and Research Hospital, Mersin Turkey; 3 Department of Gynecologic Oncology, Etlik Zübeyde Hanim Women’s Health Training and Research Hospital, Ankara Turkey

**Keywords:** Fertility-sparing treatment, endometrial cancer, endometrial intraepithelial neoplasia, reproductive outcome, survival

## Abstract

**Background/aim:**

The objective of the study was to evaluate the response, relapse, reproductive results and demographic features of the patients with endometrioid adenocancer (EAC) and endometrial intraepithelial neoplasia (EIN) who were treated with conservative treatment. This is the largest study when we consider the single center studies in this field.

**Materials and methods:**

In the current retrospective study, 38 patients (6 EAC, 31 EIN, 1 synchronous tumors of ovary and endometrium) were recruited. They were treated with progesterone products for their fertility desire and comorbidity. Reproductive results, response rates, and recurrence rates were calculated and survival analyses were performed.

**Results:**

Mean duration of the medical treatment was 10 months (range 2–60). Among the 32 patients with EIN, 28 (87.5%) had a response, 8 (25%) had a relapse and 4 (12.5%) had persistence. Among the 32 patients who expecting fertility, seven patients got pregnant (21.8%) with a total of five live births. The median follow-up was 40.5 months (range 3–180), and recurrence-free interval was 28.7 months (range 2–180).

**Conclusion:**

Fertility-sparing treatment of EAC and EIN is a feasible approach, and the eligible patients should be given a chance to get pregnant.

## 1. Introduction

Endometrial cancer (EC) is the most common gynecologic cancer whose incidence has increased in the last few decades, although mortality rates are still low [1–3]. In women of reproductive age, EC is characterized by well-differentiated endometrioid adenocarcinoma at the early stage. It has been reported that this group of patients have a more favorable prognosis than older patients [4].

 According to the World Health Organization Classification (2014), endometrial intraepithelial neoplasia (EIN) is a monoclonal precancerous lesion with atypical adenomatous hyperplasia, which can progress to EC with a rate of 29%. It also coexists with endometrial carcinoma with a rate of 48% [5].

The gold standard and curative therapy for EIN and EC is hysterectomy, whereas conservative treatment is optional for preserving fertility or to avoid morbidities or mortalities of surgery. Conservative treatment consists of medical treatment with progestins and follow-up endometrial biopsies every 3–6 months [6–8]. The most common medical treatment is high-dose oral progestins such as medroxyprogesterone acetate (MPA), megestrol acetate (MA), or levonorgestrel-releasing intrauterine device (LNG-IUD).

The fertility-sparing option takes precedence over surgical treatment in patients with EIN and EC because of the increasing number of reproductive-aged women who postpone childbearing. Fertility-sparing treatment is a well-accepted approach for patients diagnosed with endometrial intraepithelial neoplasia and early stage, grade 1, endometrioid adenocarcinoma (EAC).

 This retrospective study aimed to investigate a single center experience regarding the conservative management of patients with EIN and EC. We have analyzed the obstetric and oncologic outcomes after fertility-sparing treatment of EIN and EAC and also responses to the treatment.

## 2. Materials and methods

In this retrospective study, we recruited 38 patients in the current study, of which a total of 6 were with EAC, one was with co-occurrence of the EAC of the ovary and EIN and 31 of all were with EIN. The clinical files of all patients with EIN/EC who were treated conservatively at the University of Health Sciences Etlik Zübeyde Hanım Gynecologic Oncology Clinic were reviewed between March 2005 and May 2020. Eligible patients who were included in the study were aged between 21 and 88 years old and had histopathologically confirmed EIN according to the WHO 2014 Classification of Endometrial Hyperplasias and Grade 1 EC and Grade 2 EC according to the 2009 International Federation of Obstetrics and Gynaecology Staging System. The following information was obtained from the patients’ charts: age, body mass index, parity, type and duration of infertility, comorbidities, diagnostic methods, histopathological diagnose, duration and dose of progestin treatment, presence of complete response or recurrence, duration of follow-up, method of conception. All of the patients were fastidiously informed about the risks of the existing disease and conservative treatment. The study was performed with the permission of the Training Plan and Coordination Board Committee of our institution (18/06/2019- No: 10).

Patients were divided into three groups according to their histopathological results: EIN, EAC Grade 1, and EAC Grade 2. First diagnosis was determined with endometrial tissue sampling by probe curettage in all patients. Dilatation curettage (D&C) was then performed to the patients to not miss out any upstage pathology. Transvaginal sonography was routinely performed for the presence of any adnexal masses. In the patients with a diagnosis of endometrial cancer, magnetic resonance was performed to identify any extension of endometrioid adenocarcinoma or myometrial invasion before the beginning of medical treatment. Medical treatment based on progestin therapy was used for the initial treatment. 

If the disease is progressive, a total hysterectomy with bilateral salpingo-oophorectomy procedure was strongly recommended. Complete response was defined as the absence of disease on follow-up endometrial curettages. Recurrence was defined as the detection of EC or EIN during the 3- or 6-months later follow-up endometrial sampling following an endometrial sample result that showed disease regression. Time to recurrence was calculated from the date of complete regression. Persistence was defined as the presence of the initial pathology on follow-up endometrial curettages. Live births were defined as the birth of healthy infants, and its rate was defined as the ratio of the women who gave birth to healthy infants divided by the total number of women undergoing fertility-sparing therapy. After a regression achievement, patient who desired fertility was directed to the infertility department. 

Statistical data analysis was performed using the Statistical Package for Social Sciences version 21 (IBM Corp., Armonk, NY, USA). The categorical data was performed using descriptive statistical methods. Variations between the unpaired groups were analyzed using the Mann–Whitney U test. Cox proportional hazards regression analysis method was used to investigate the univariate effects of BMI, age, histopathology, response and relapse on survival. Recurrence free survival (RFS) rates of the patients were calculated from the date of the complete response to the date of recurrence, and overall survival (OS) rates of the patients were calculated from the date of diagnosis to the date of death or last follow-up. RFS and OS were estimated by using the Kaplan–Meier method. All p values <0.05 were considered significant. 

## 3. Results

### 3.1. Patient characteristics

In the current study, 38 patients who underwent medical treatment for EIN and EAC were analyzed. The mean age of the patients that underwent conservative treatment (both fertility sparing and comorbidities) was 34.78 years (range, 21–88). There were six patients with comorbidities or who did not have fertility desire. One of them was in late-stage severe dementia due to Alzheimer’s disease (88 year old patient), the other had hypophysis tumor (30 year old patient), and the rest of them did not want to be pregnant and have hysterectomy because of their young age (23, 33, 34 and 36 years old, respectively). The mean value of the BMI was 32.85 kg/m^2^ (range 20–48). In all, 28 (73.6%) patients were diagnosed via probe curettage, 6 (15.7%) were diagnosed via hysteroscopy and biopsy, and 4 were diagnosed via dilatation and curettage (10.5%). In total, 32 (84.2%) patients have been diagnosed with EIN, and, among these patients, one of them had a co-occurrence of the EAC of the ovary and EIN. Rest of the patients had a diagnosis of EAC; 4 (10.5%) had grade 1, and 2 (5.3%) had grade 2 disease, respectively. The patients’ characteristics, including medical treatments, are shown in Table 1. 

**Table 1:** Characteristics of patients who underwent conservative treatment.

Characteristics	Patients (n = 38)
Age (year)	34.78 (21–88)
BMI (kg/m2)	32.85 (20–48)
<25	4 (10.5%)
≥25, <30	9 (23.7%)
≥30, <35	11 (28.9%)
≥35, <40	5 (13.2%)
≥40	9 (23.7%)
History of infertility	32
Primary	22 (68.75%)
Secondary	10 (31.25%)
Diagnose tool	
D&C	4 (10.5%)
H/S Bx	6 (15.7%)
P/C	28 (73.6%)
Histopathology	
EIN	32 (84.2%)
EAC Grade 1	4 (10.5%)
EAC Grade 2	2 (5.3%)
Initial medical treatment	
Megestrol acetate	33 (86.8%)
Micronized progesterone	3 (7.9%)
MPA	2 (5.3%)

BMI: body mass index, D&C: dilatation curettage, H/S bx: hysteroscopic biopsy, P/C: probe curettage, EIN: endometrial intraepithelial neoplasia, EAC: endometrioid adenocancer, MPA: medroxyprogesterone acetate.

### 3.2. Evaluation of the treatment

All in all, 33 (86.8%) patients were treated with megestrol acetate, 3 (7.9%) of them were treated with micronized progesterone, and 2 (5.3%) patients were treated with medroxyprogesterone acetate (MPA) at the time of the initial diagnosis (Table 1). Megestrol acetate was the most commonly used drug, with a daily dose range of 80–480 mg (mostly 160 mg) and a mean treatment duration of 10.4 months (range 2–60). Micronized progesterone was the second commonly preferred drug with a dose of 200–400 mg/daily, and treatment durations were 2, 6 and 9 months. MPA was administered in two patients with a 10mg/daily dose, and the treatment durations were 2 and 12 months, respectively. 

One of the patients who have treated with megestrol acetate changed into Levonorgestrel intrauterine system (LNG-IUD) because of relapse. Mean duration of the medical treatment was 10 months (range 2–60). Among the 32 patients with EIN, 28 (87.5%) had a response, 8 (25%) had a relapse, and 4 (12.5%) had persistence (Table 2). Interestingly, there were 4 patients with persistence and without response in the EAC G1 group. All of the patients with EAC G2 have shown response to the treatment except one patient with a relapse (Table 2).

**Table 2:** Outcome of the treatment according to the histopathologic group.

Histopathology	Response	Relapse	Persistence	Pregnancy	Live birth
EIN (32)	28	8	4	7	5
EAC Grade 1 (4)	0	1	4	0	0
EAC Grade 2 (2)	2	1	0	0	0
Total	30(total of 38, 78.9 %)	10(total of 33, 30.3 %)	8(total of 33, 24.2%)	7(total of 32, 21.8 %)	5(total of 32, 15.6%)

 Among the 32 patients who expected fertility, seven patients got pregnant (21.8%) with a total of five live births (Table 2). Only one of them got pregnant with artificial reproductive techniques, whereas the others got pregnant spontaneously/in natural ways. All of the pregnancies were seen in the EIN group. 

**Figure 1:**
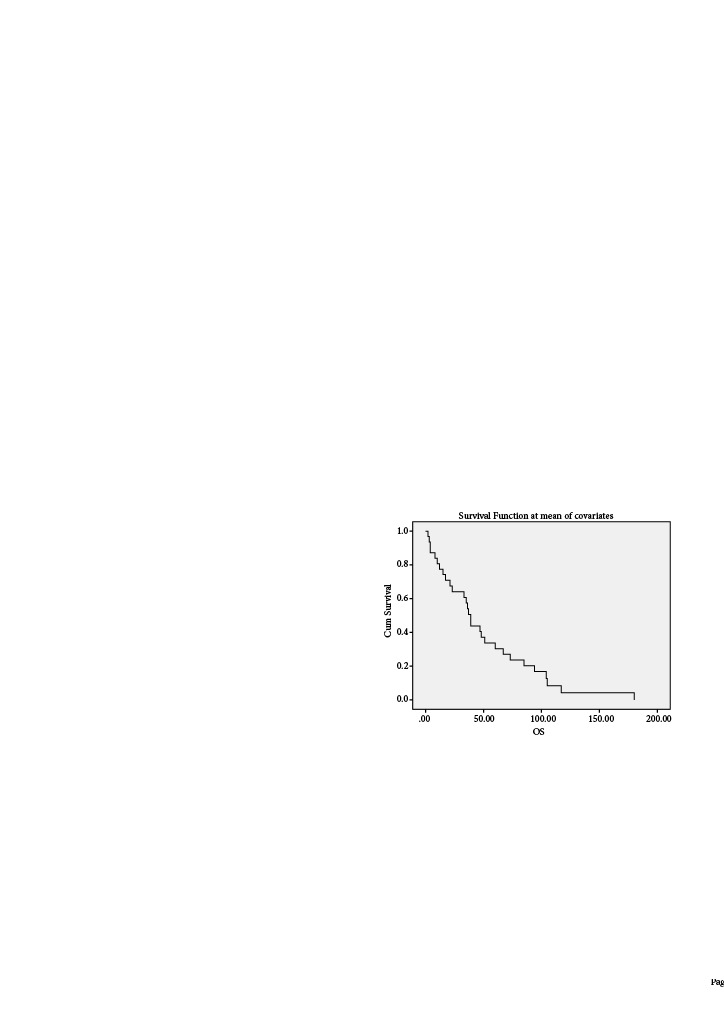
Survival analysis of the patients.

**Figure 2:**
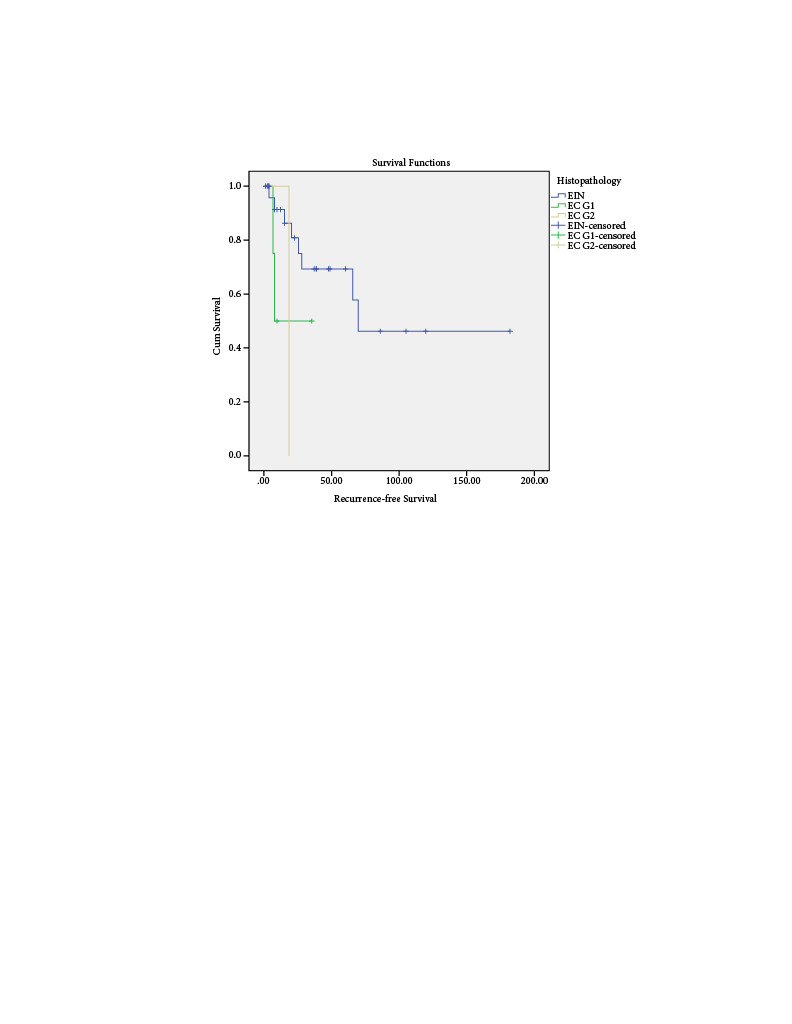
Recurrence free survival according to the histopathologic groups.

The median follow-up was 40.5 months (range 3–180), and recurrence-free interval was 28.7 months (range 2–180, Figure 1 and Figure 2). According to the Kaplan–Meier method, the cumulative overall survival rate was 91%, and the cumulative recurrence free survival rate was 62%. Ten patients underwent definitive surgery, whereas 28 patients (73.6%) still have their uterus today. On the basis of the pathological findings after the surgeries, there were two patients with advanced endometrioid carcinoma (stage IIIC2 according to the FIGO 2009) whose initial pathologies were EAC G1 and EIN (Table 3). Their status resulted in an exitus. Among the four exitus in our series, the rest two (EAC G1 and EIN) were caused by uncertain reasons that are not related to the current disease. One of these two patients was 88 years old patient who received megestrol acetate 160 mg for nine months and died at the end of the nine months because of an uncertain reason. One patient who underwent a debulking operation with left salphingo-oophorectomy, pelvic/paraaortic lymph node dissection, infracolic omentectomy and endometrial biopsy had an endometrioid type grade 1 ovarian cancer, stage IC2 according to the FIGO 2014 ovarian cancer classification system, co-occurrence of EAC G1 endometrial biopsy pathology, whereas the initial pathology was EIN. She is still alive and had a pregnancy resulting in abortus. 

**Table 3:** Results and outcomes of the operations.

Initial pathology	Operation	Postoperative pathology	Stage	Pregnancy	Status
EIN	TAH+BSO	Malignite negative	None	Live birth	Alive
EIN	TAH+BSO	EAC G1	IA	None	Alive
EIN	TAH+BSO	EAC G1	IA	None	Alive
EIN	TAH+BSO	EAC G1	IA	None	Alive
EIN	TAH+BSO	EIN	None	None	Alive
EIN	Left USO+BPPLND+Omentectomy+ D&C	Endometrioid type Grade 1 ovarian cancer + EAC G1	IC2 Ovarian cancer+ G1 EAC	None	Alive
EIN	TAH+BSO+BPPLND+Omentectomy	EAC G3	IIIC2	None	Exitus
EAC G1	TAH+BSO	EAC G1	IA	None	Alive
EAC G1	TAH+BSO	EAC G1	IA	None	Alive
EAC G1	TAH+BSO+BPPLND+Omentectomy	EAC G3	IIIC2	None	Exitus

TAH+BSO:Total Abdominal Hysterectomy+Bilateral Salphingo-oophorectomy

## 4. Discussion

In the current study, our aim was to analyze the results of the conservative treatment of the patients with EIN/EAC who desire fertility or have comorbidities. 

 Although there are limited data available on prospective conservative treatments, fertility preserving treatment in eligible patients with cancerous and precancerous pathologies is an accepted approach today just because a great amount of endometrial cancer is characterized by well differentiated endometrioid carcinoma and at early stage [4, 9,10]. 

Progestin treatment is the most well accepted approach for the conservative treatment, whereas there is no sufficient evidence to specify the convenient dose and duration of treatment. Oral progestogens such as MPA and MA are the most preferable materials, but, besides these, LNG-IUD is an option for a local effect to the endometrial tissue [11]. LNG-IUD has been shown as a sufficient and an influential option for the treatment in some studies [12,13]. In the present study, megestrol acetate was the most commonly preferred drug with a range of 80–480 mg dose, daily (mainly 160 mg). 

Fan et al. [14] performed an extensive metaanalysis that contains 28 study and 619 cases of early stage endometrial cancer were analyzed to evaluate the efficacy of conservative treatment. The authors reported that 76.3% patients showed remission, whereas 30.7% have recurrence rate. In another metaanalysis, Qin et al. [15] reported that 82.4% of patients showed a response to hormonal therapy addition with a relapse rate of 25.0%. On the other hand, according to our study, the response rate and the recurrence rate were 78.9% and 30.3%, respectively. Similar to the present study, Kim et al. [16] demonstrated a response rate of 80% in EC patients treated with progestins.

One of the most important goals of fertility preservation is to achieve pregnancy. The National Comprehensive Cancer Network and European Society of Gynaecological Oncology (ESGO) confirms that fertility sparing treatment is a safe option for stage IA, grade 1 EC patients with endometrioid type and which disease is limited to endometrium [17]. When we look at the literature and compare the results of the present study, we can see that the pregnancy rate after complete remission is about 30% [18]. In our study, pregnancy rate and live birth rate were 21.8% and 15.6%, respectively. Qin et al. reported a pregnancy rate of 28.8%. The live birth rate of this metaanalysis was 19.6% [15]. The majority of the pregnancies obtained by assisted reproductive techniques (ART) are opposed to the current study.

Gonthier et al. defined in a multicenter study that BMI of 30 kg/m^2^ or greater was associated with a lower probability of pregnancy [19]. In our study, 25 of all (65%) patients had BMI of 30 kg/m^2^ or greater, so this would be related to a lower pregnancy rate. 

The surgical procedure is another hot point in these patients after completion of fertility desire. The question is whether to preserve or not the ovaries in young patients with EIN or EAC, regarding the surgical approach after the recurrence. In the present study, one patient who was 25 years old had synchronous ovarian cancer (G1 EA) and G1 EC of endometrium. In a recent study by Wang et al., 1 patient had ovarian metastasis, and 3 patients were found to have synchronous ovarian cancer (G1EA) [1]. However, previous studies proposed that synchronization of endometrial and ovarian carcinoma does not worsen the survival and prognostic pattern [19–22]. 

Women undergoing conservative therapy should be perplexed about their chances of survival. Park et al. [9] demonstrated that 85% of patients showed disease regression with oral progestin treatment within a follow-up duration of 51 months (range, 24–160 months). These findings support the fact that progestin treatment should be recommended to patients who have a desire to preserve fertility. 

In the current study, we analyzed the results of the conservative treated patients. There were a few limitations that should be indicated. First, there was a small number of patients although the number was enough for a single center study. Second, this was a retrospective study that was based on clinical data. The strengths of the study are that its relatively large sample size and analyses of the outcomes. This work claims that medical treatment is a feasible approach in early stage (G1 and no myometrial invasion) endometrioid cancer and EIN patients with comorbidities and fertility expectations. Also, a chance should be given to the spontaneous pregnancies over ART. Further prospective studies with a high patient population are needed to make clear the treatment efficacy.

To the best of our knowledge, this is the largest single center study with regards to the number of patients with EIN. Fertility-sparing treatment of EAC and EIN is a feasible approach, and a chance should be given to spontaneous pregnancies together with artificial reproductive techniques.

## Authors’ contribution 

Concept/design: Nurettin BORAN, Fulya KAYIKCIOGLU Data collection and processing: Esra ISCI BOSTANCI, Yasin DURMUS, A.Sinem DURU COTELI Analysis and interpretation: Yasin DURMUS Writing manuscript: Esra ISCI BOSTANCI Critical review: Nurettin BORAN, Esra ISCI BOSTANCI
